# Gastric schwannoma: a benign tumour often mistaken clinically, radiologically and histopathologically for a gastrointestinal stromal tumour – a case series

**DOI:** 10.1308/003588412X13171221590935

**Published:** 2012-05

**Authors:** JML Williamson, MS Wadley, NA Shepherd, S Dwerryhouse

**Affiliations:** ^1^Department of Oesophagogastric Surgery, Gloucestershire Royal Hospital, Great Western RoadGloucester, GLI 3NN; ^2^Department of Upper GI Surgery, Worcestershire Royal Hospital, Charles Hastings WayWorcester Worcestershire, WR5 1DD; ^3^Gloucestershire Cellular Pathology Laboratory, Cheltenham General Hospital, CheltenhamGlos, GL53 7AN

**Keywords:** Schwannoma, Stomach, Nerve sheath tumor, S100 protein

## Abstract

**INTRODUCTION:**

Gastric schwannomas are rare mesenchymal tumours that arise from the nerve plexus of the gut wall. They present with non-specific symptoms and are often detected incidentally. Pre-operative investigation is not pathognomonic and many are therefore diagnosed as gastrointestinal stromal tumours (GISTs). Operative resection is usually curative as they are almost always benign, underpinning the importance of differentiating them from GISTs.

**METHODS:**

Three cases of gastric schwannomas were identified over a seven-year period. The clinical details and management were reviewed retrospectively.

**RESULTS:**

There were two women and one man with a mean age of 62 years (range: 51–69 years). Two patients presented with bleeding and one with abdominal pain. The mean tumour size was 5.2cm (range: 2–10cm) and the tumours were resected completely following total or wedge gastrectomies. Histology in all cases showed spindle cells with a cuff of lymphoid tissue. Immunohistochemistry confirmed positive S100 staining and negative CD117 and DOG-1 staining in all cases.

**CONCLUSIONS:**

We report our experience with these unusual primary stromal tumours of the gut and their presentations, preoperative investigations, operative findings and pathological findings are discussed. Operative resection in all cases has been considered curative, which is supported by previous series confirming the excellent prognosis of gastric schwannomas.

Gastric schwannomas are rare, comprising 0.2% of all gastric tumours.^1^ They can occur at any age but are most frequently noted in the fifth decade with a female predominance.^1^^–^^6^ Symptoms are non-specific and related to site (eg epigastric mass), size (eg pain) and local invasion (eg gastrointestinal bleeding). A significant proportion of lesions are detected incidentally during routine abdominal investigation. Computed tomography (CT) and upper gastrointestinal endoscopy are the mainstays of investigation although neither of these investigations is pathognomonic. Schwannomas are misdiagnosed frequently as gastrointestinal stromal tumours (GISTs), with their true nature only revealed by accurate histological and immunohistochemical analysis.^2^ Indeed, histologically, they may be mistaken for GISTs as they tend to lack the characteristic morphological features of schwannomas seen elsewhere (especially the skin) but do have important histological differentiating features. Immunohistochemistry is required, however, to accurately type all such stromal tumours in the gut.

Gastric schwannomas are almost uniformly benign and surgical resection therefore offers excellent prognosis. This is in distinct contrast to the major differential diagnosis clinically, endoscopically, radiologically and histopathologically, the GIST. We describe three cases of gastric schwannomas that underwent operative resection. The aim of this paper is to report our experience of these unusual tumours, highlighting diagnostic adjuncts, operative strategies and histological findings.

## Methods

Three patients with a histological diagnosis of gastric schwannomas were identified in one cancer network over a seven-year period (2004–20H). The clinical details and management of each case were analysed retrospectively. These details are summarised in Table 1.

**Table 1:** Summary of clinical details and management of the three patients with gastric schwannomas (positive/negative staining marked as +ve/-ve)

Case	Age/sex	Presenting symptom	Investigations	Tumour size / location	Treatment	Histology	Immunohistochemistry
1	51F	Pain	US, CT, OGD, EUS, PET	10cm, lesser curve	Total gastrectomy	Spindle cells, lymphoid cuff	S100 +ve, CD117 -ve, DOG-1 -ve, smooth muscle actin -ve, desmin -ve CD34 -ve
2	69F	Melaena	OGD, EUS, CT	2cm, greater curve	Wedge gastrectomy	Spindle cells, lymphoid cuff	SlOO +ve, CD117 -ve, DOG-1 -ve
3	66M	Anaemia	OGD, EUS, CT	3.5cm, posterior wall	Wedge gastrectomy	Spindle cells, mast cells, lymphoid cuff	S100 +ve, CD117 -ve, DOG-1 -ve

US = ultrasonography; CT = computed tomography; OGD = oesophagogastroduodenoscopy; EUS = endoscopic ultrasonography; PET = positron emission tomography

## Case 1

**Figure 1:**
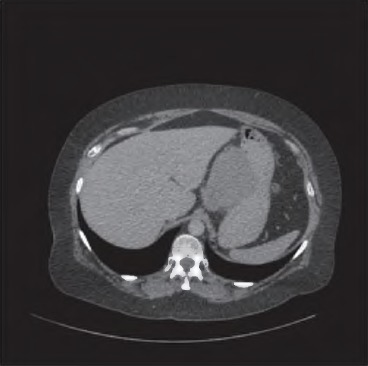
Computed tomography showing the 9cm irregular homogenous lesion arising from the lesser curve of the stomach

A 51-year-old woman was investigated for atypical right upper quadrant pain and mildly elevated serum aspartate transaminase levels. Abdominal ultrasonography detected an incidental epigastric mass. CT revealed a 9cm x 6cm irregular homogenous lesion arising from the lesser curve of the stomach (Fig 1) and two coeliac axis lymph nodes. Gastroscopy demonstrated no mucosal lesion and endoscopic ultrasonography (EUS) suggested a GIST as a likely diagnosis but EUS fíne needle aspiration of the lesion was not diagnostic. Positron emission tomography (PET) revealed avid uptake in the tumour but no uptake in the lymph nodes.

**Figure 2:**
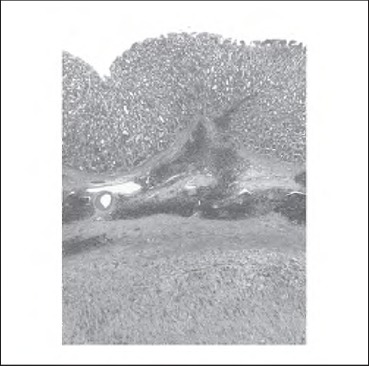
Low power view of the tumour with normal body-type gastric mucosa above and the prominent lymphoid cuff between

**Figure 3:**
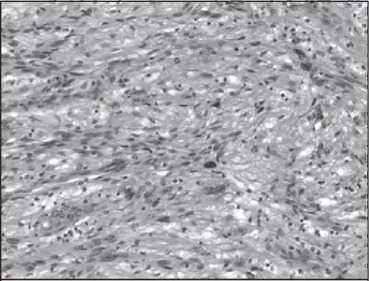
High power view of the tumour showing typical features of a spindle cell tumour with moderate nuclear pleomorphism but no mitotic activity

At laparotomy, a 10cm lesion arising from the upper half of the lesser curve that was encasing her left gastric vessels was found and a total gastrectomy was performed. Histological examination revealed a benign gastric schwannoma with haphazardly arranged spindle cells and a cuff of lymphoid tissue peripherally (Figs 2 and 3). Immunohistochemistry was positive for S100 and negative for CDH7, DOG-1, smooth muscle actin, desmin and CD34.

## Case 2

**Figure 4:**
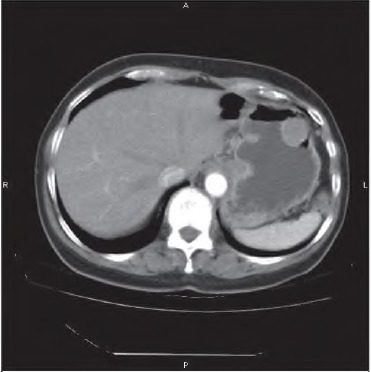
Computed tomography showing a 2cm submucosal lesion in the mid body of the stomach

**Figure 5:**
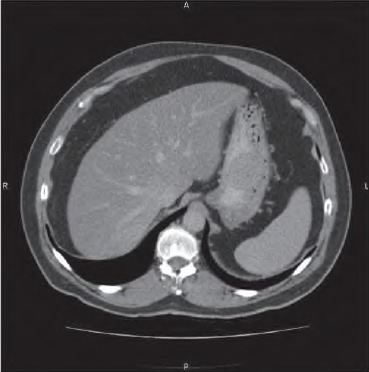
SlOO immunohistochemistry: The tumour cells are strongly positive as is a small peripheral nerve entrapped in the capsule of the tumour (central)

A 69-year-old woman underwent gastroscopy for melaena and anaemia. A 3cm ulcerated tumour was identified on the greater gastric curve that was clinically suspicious of a GIST. Biopsy of the lesion was not diagnostic, revealing only inflammatory cells, and EUS demonstrated a well demarcat ed, submucosal lesion that did not breach the serosal layer. CT confirmed the location and size of the tumour (Fig 4), with no evidence of local invasion or métastasés. At laparoscopy, a 2cm serosal tumour was identified in the mid body of the stomach on the anterior wall. A wedge resection of the tumour was performed with the use of an endoscopic linear stapler. Pathological investigation revealed a 3cm well circumscribed, dumbbell-shaped submucosal tumour. Histology revealed that the tumour comprised spindle cells and was surrounded by a lymphoid ring. Immunohistochemistry demonstrated the tumour cells to be S100 positive (Fig 5) and CDH7 and DOG-1 negative.

## Case 3

**Figure 6:**
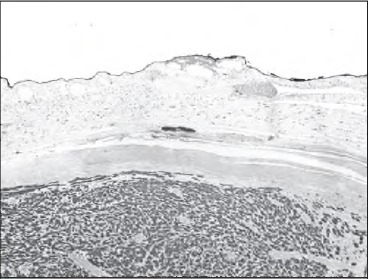
Computed tomography showing a 5cm submucosal homogenous lesion arising from the fundus of the stomach

A 66-year-old man was investigated for anaemia with melaena and haematemesis with an upper gastrointestinal endoscopy. At gastroscopy, a 5cm submucosal lesion was identified with an area of central ulceration. It was located on the anterior gastric wall, approximately 5cm from the oesophagogastric junction. EUS measured the lesion at 5.8cm and illustrated that it had a 50% intraluminal component but there were no features of local invasion, lymphadenopathy or distal spread. Abdominal CT confirmed the absence of any metastatic spread and showed the lesion to be amenable to laparoscopic resection (Fig 6).

At laparoscopy, a 4cm anterior tumour was seen and ontable endoscopy confirmed a 50% intragastric component. The tumour was excised in a wedge via an endoscopic stapler. Pathological examination confirmed a 55mm ulcerated tumour that was composed of spindle cells and mast cells. The tumour was surrounded by a florid lymphoid infíltrate. It was negative for CDH7 and DOG-1 staining and positive for S100 staining on immunohistochemistry.

## Discussion

Gastric schwannomas represent 2–7% of all gastrointestinal mesenchymal tumours (the most common types being GISTs and smooth muscle tumours).^2^^–^^4^ They are thought to arise from the nerve plexus of the gut wall, in contrast to ‘conventional’ schwannomas, which arise from peripheral nerves in the skin, connective tissues and other internal organs.^5^^–^^7^

Gastric schwannomas can occur at any age but are most frequently noted in the fifth decade with a female predominance.^1^^–^^6^ They cause non-specific symptoms of pain and an epigastric mass although many are detected incidentally.^4^ Symptoms are thought to relate to the tumour size (diameters of 0.5–15.5cm have been reported) rather than local invasion.^2^^–^^4^ Since being first described in 1988 by Daimarau *et al,^6^* there has only been one reported malignant case of a gastric schwannoma^8^ and one related to von Recklinghausen’s disease.^4^

Definitive pre-operative diagnosis can be difficult to establish due to the non-specific findings seen on cross-sectional imaging and endoscopy.^2^, ^9^ This is in part due to their rarity and limited data in the literature.^2^ Moreover, endoscopic biopsy often fails to obtain a representative sample as the lesions originate deep to the gastric mucosa and superficial biopsies will not usually contain tumour.^2^ Gastric schwannomas are therefore frequently reported clinically, endoscopically and radiologically as GISTs, as in our patients.

CT shows that schwannomas have a homogenous appearance while the majority of GISTs (87–92%) are heterogeneous (often due to haemorrhage and necrosis within them).^10^, ^11^ Ultrasonography, magnetic resonance imaging and PET have all been used to evaluate gastric lesions but, again, features are non-specific for schwannomas.^2^, ^4^ EUS may reveal a marginal halo, corresponding to a lymphoid cuff, around the tumour.^12^ Pre-operative imaging should establish the extent of the disease and any local invasion or displacement by the tumour.

Tumours are usually described as having a firm or rubbery consistency with a homogenous tan-yellow or whitish cut surface.^2^, ^4^ Histological appearances are of well circumcised lesions typically surrounded by a cuff of lymphoid aggregates.^2^, ^6^, ^9^, ^12^ The latter feature is highly characteristic and almost pathognomonic of gastrointestinal schwannomas and yet it is most unusual in schwannomas at other sites and not seen in the major differential diagnoses, such as GISTs, inflammatory fibroid polyp and other stromal tumours. The tumours are highly cellular and comprise mainly bipolar spindle cells.^9^

Another highly characteristic feature is that they lack the typical Antoni A and Antoni B areas of schwannomas seen elsewhere and may have pronounced nuclear pleomorphism (but very low mitotic activity). This is because many of these tumours are discovered incidentally and have been present for some time. They tend to therefore show features more characteristically associated with so-called ancient schwannomas as described above. Immunohistochemistry reveals positive staining for S100 but not for CD45, CDH7 or DOG-1, which helps distinguish them from GISTs.^4^, ^9^ All of our cases showed these histological and immunohistochemical findings.

Operative resection should follow the same principle as for GIST, including the lesion and any involved adjacent structures, with the goal of achieving an R0 resection. The extent of gastric resection is dependent on the size and location of the tumour as well as the presence of any invasion of local structures. Resection can be achieved via an open or laparoscopic approach. Reported outcomes are excellent given the predominance to benign disease.^3^, ^5^^–^^7^, ^13^ Routine long-term follow-up should therefore not be offered and the operation should be considered curative unless any signs of malignant transformation are identified.

## Conclusions

Our cases illustrate the spectrum of presentation of gastric schwannomas. Moreover, they highlight how these unusual tumours may be misdiagnosed pre-operatively (and potentially histopathologically unless appropriate immunohistochemistry is undertaken) despite both radiological and endoscopic assessment. Pathological investigations were consistent with the published literature and operative resection should be considered curative as these tumours are benign.
